# Long non-coding RNA SNHG20 promotes non-small cell lung cancer cell proliferation and migration by epigenetically silencing of P21 expression

**DOI:** 10.1038/cddis.2017.484

**Published:** 2017-10-05

**Authors:** Zhenyao Chen, Xin Chen, Ping Chen, Shanxun Yu, Fengqi Nie, Binbin Lu, Te Zhang, Yue Zhou, Qinnan Chen, Chenchen Wei, Wei Wang, Zhaoxia Wang

**Affiliations:** 1 Department of Oncology, Second Affiliated Hospital, Nanjing Medical University, Nanjing, People’s Republic of China; 2 Cancer Medical Center, Second Affiliated Hospital of Nanjing Medical University, Nanjing, People’s Republic of China; 3 First Clinical College of Nanjing Medical University, Nanjing, People’s Republic of China; 4 Department of Thoracic Surgery, First Affiliated Hospital, Nanjing Medical University, Nanjing, People’s Republic of China

## Abstract

Mounting evidence demonstrates that long non-coding RNAs (lncRNAs) are novel transcripts governing multiple biological processes, and their dysregulation is involved in the development and progression of multiple types of cancers. Small Nucleolar RNA Host Gene 20 (SNHG20) is a 2183 bp lncRNA, and its overexpression predicts poor prognosis in colorectal cancer and hepatocellular carcinoma. However, the clinical relevance of SNHG20 and its molecular mechanisms affecting cancer cell phenotype have not been documented. Here, we found that SNHG20 was upregulated in non-small cell lung cancer (NSCLC) tissues compared with normal samples. Higher SNHG20 expression was significantly associated with advanced tumor, lymph node and metastases (TNM) stage and tumor size, as well as poorer overall survival. Moreover, knockdown of SNHG20 repressed NSCLC cell proliferation, migration and induced cell apoptosis. Mechanistic investigations revealed that SNHG20 could interact with EZH2 (enhancer of zeste homolog 2), thereby repressing P21 expression. Furthermore, rescue experiments indicated that SNHG20 functioned as an oncogene partly via repressing p21 in NSCLC cells. Taken together, our findings demonstrate that SNHG20 is a new candidate for use in NSCLC diagnosis, prognosis and therapy.

Lung cancer is one of the most common causes of cancer-associated death worldwide. Non-small cell lung cancer (NSCLC), including adenocarcinoma, squamous cell carcinoma and large cell carcinoma, accounts for approximately 80–85% of all lung cancer cases. In spite of recent progress in clinical diagnosis and treatment for NSCLC, the overall survival (OS) time of NSCLC patients has not substantially improved, and the 5-year survival rate remains below 15%,^1, 2^ which is because most NSCLC patients are diagnosed at an advanced stage and with metastasis. Therefore, understanding the detailed molecular mechanism for NSCLC and identifying novel biomarkers is necessary for early diagnosis, prevention and treatment.

With the rapid development of high-throughput sequencing and microarray techniques, an increasing number of non-coding genes have recently been discovered.^3, 4^ These thousands of non-coding genes yield non-coding RNAs, such as microRNAs and long non-coding RNAs (lncRNAs). lncRNAs are longer than 200 nt, and were once considered to be cloning artifacts or transcription noise because of their lack of protein-coding ability. However, recent studies have revealed that numerous lncRNAs are expressed in different human tissues, and they are expressed in spatially and temporally specific patterns.^5^ These lncRNAs participate in multiple cellular processes, such as X chromosome imprinting, muscle cell differentiation, cell fate decisions, the immune response, epithelial–mesenchymal transition, cancer cell growth, metastasis and drug resistance.^6, 7, 8, 9, 10, 11, 12, 13^ Importantly, emerging evidence has demonstrated that dysregulation of lncRNAs is involved in a number of diseases,^14, 15^ including NSCLC.^16, 17, 18, 19^ For example, our previous studies found that overexpression of the lncRNA, PVT1, promotes NSCLC cell proliferation through interaction with enhancer of zeste homolog 2 (EZH2), and thereby represses large tumor suppressor kinase 2 (LATS2) expression.

Small nucleolar RNAs (snoRNAs) are lncRNAs that are increasingly considered as important regulators of protein synthesis. With the rapid progress in lncRNA research, the exploration of formerly unappreciated non-protein-coding genes, such as snoRNAs, may reveal their various biological effects and molecular mechanisms. In addition, recent studies indicate that snoRNAs might play crucial roles in carcinogenesis and progression of cancer.^20, 21, 22^ Siprashvili *et al.*^23^ reported that SNORD50A and SNORD50B could directly bind K-Ras and were recurrently deleted in human cancer. Small nucleolar RNA host gene 20 (SNHG20), 2183 nt in length, is located on 17q25.2. and is involved in colorectal cancer and hepatocellular carcinoma,^24, 25, 26^ but its clinical relevance and functional roles in NSCLC are still unknown. In the present study, we found that expression of SNHG20 was upregulated in NSCLC tissues compared with adjacent normal tissues. Increased SNHG20 expression was positively correlated with poorer prognosis for NSCLC patients. Furthermore, we demonstrated that aberrant expression of SNHG20 promoted cell proliferation and migration in NSCLC cells, partly via binding with EZH2 to transcriptionally downregulate P21 expression.

## Results

### SNHG20 expression was upregulated in human NSCLC tissues and correlated with poor prognosis

**Figure 1:**
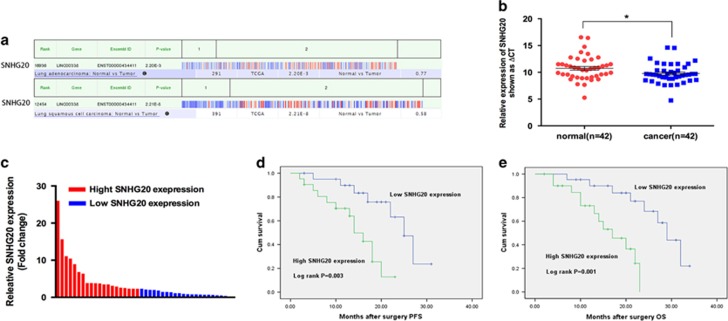
Expression of SNHG20 in NSCLC tissues and its clinical significance. (**a**) Relative expression of SNHG20 (also known as LINC00338) in NSCLC tissues compared with normal tissue was analyzed using the Cancer Genome Atlas data set. (**b**) SNHG20 expression levels in NSCLC tissues (*n*=42) compared with corresponding non-tumor tissues (*n*=42) were examined by qRT-PCR and normalized against GAPDH expression. The data are presented as the delta CT value. (**c**) The patients were classified into two groups according to SNHG20 expression. (**d** and **e**) Kaplan–Meier progression-free survival and overall survival curves according to SNHG20 expression levels. Data are presented as the mean±S.E.M. *P<0.05, **P<0.01

In this study, we first analyzed the expression levels of SNHG20 in human NSCLC tissues using sequencing data downloaded from the Cancer Genome Atlas (TCGA), and found that SNHG20 expression levels were significantly upregulated in NSCLC tissues compared with adjacent histologically normal tissues (Figure 1a). Furthermore, SNHG20 expression levels were determined in 42 paired NSCLC samples and normal counterparts using qRT-PCR assays and normalized to GAPDH (*P*<0.05; Figure 1b). These results were consistent with the TCGA sequencing data. Moreover, to assess whether SNHG20 was differentially expressed in NSCLC tissues, 42 NSCLC patients were classified into two groups relative to the median ratio of SNHG20 expression in tumor tissues: relative high SNHG20 group (*n*=21, SNHG20 expression ratio≥median ratio) and relative low SNHG20 group (*n*=21, SNHG20 expression ratio≤median ratio) (Figure 1c). Clinicopathological characteristics of the 42 primary NSCLC patients are summarized in Table 1. Notably, statistical analysis revealed that high SNHG20 expression levels in NSCLC were significantly correlated with larger tumor size (*P*=0.012), lymph node invasion (*P*=0.005) and TNM stage (*P*=0.008). However, there was no significant correlation between SNHG20 expression and other features such as age (*P*=0.758) and sex (*P*=0.756) in NSCLC.

### Association between SNHG20 expression and patient survival

To further evaluate the relationship between SNHG20 expression levels and NSCLC patients' outcomes, we used Kaplan–Meier survival analysis and the log-rank test. Progression-free survival (PFS) and OS curves were plotted according to SNHG20 expression levels. The results showed that the PFS rate over 3 years for cases with high SNHG20 expression was 38.1%, while it was 66.7% for low SNHG20 expression cases. Median survival time for the high SNHG20 group was 14 months, and 23 months for the low SNHG20 group (Figure 1d). As shown in Figure 1e, the OS rate over 3 years for cases with high SNHG20 expression was 42.9%, but was 66.7% for low SNHG20 expression cases. Finally, the median survival time of cases with high SNHG20 expression was 16 months, but 27 months for cases with low SNHG20 expression. Remarkably, patients with high SNHG20 expression levels had poorer PFS (*P*=0.003) and OS (*P*=0.001). These results indicate that overexpression of SNHG20 may represent a novel indicator of poor prognosis or a progression marker for NSCLC.

### Modulation of SNHG20 expression in NSCLC cells

**Figure 2:**
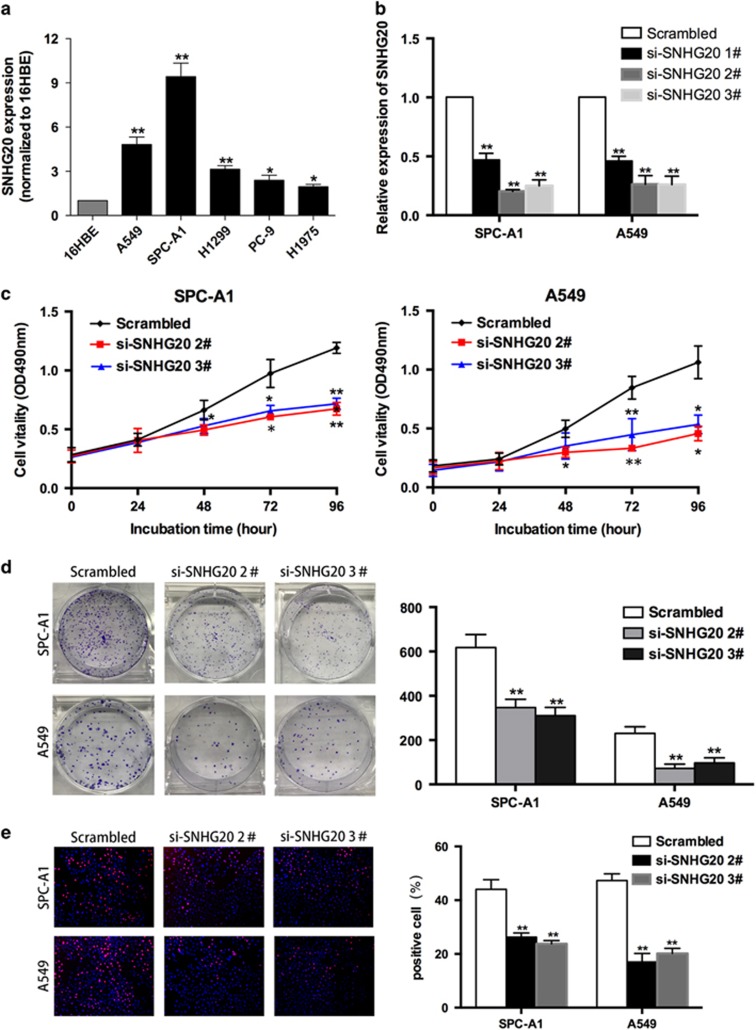
Effects of SNHG20 on NSCLC cell proliferation *in vitro*. (**a**) qRT-PCR analysis of SNHG20 expression in normal human bronchial epithelial cells (16HBE) and NSCLC cells. (**b**) qRT-PCR analysis of SNHG20 expression in control (scrambled), si-SNHG20 1#, si-SNHG20 2# and si-SNHG20 3# treated NSCLC cells. (**c**) MTT assays were used to determine the viability of si-SNHG20-transfected SPC-A1 and A549 cells. (**d**) Colony formation assays were performed to determine the proliferation of si-SNHG20-transfected SPC-A1 and A549 cells. Colonies were counted and captured. (**e**) EdU staining assays were performed to determine the growth of si-SNHG20 transfected SPC-A1 and A549 cells. Representative images and data are based on three independent experiments. Data are presented as the mean±S.E.M. *P<0.05, **P<0.01

To investigate the functional role of SNHG20 in NSCLC cells, we first performed qRT-PCR analysis to examine the expression of SNHG20 in a diverse range of human NSCLC cell lines. As shown in Figure 2a, SNHG20 expression was significantly upregulated in two NSCLC cell lines (SPC-A1 and A549) compared with that in a normal human bronchial epithelial cell line (16HBE). Next, we designed three different SNHG20 siRNAs to transfect the two NSCLC cell lines. qRT-PCR analysis was performed at 48 h post-transfection and revealed that si-SNHG20 2# and 3# had higher efficiency of interference than si-SNHG20 1# (Figure 2b). Therefore, we selected si-SNHG20 2# and 3# for subsequent experiments.

### Knockdown of SNHG20 inhibits NSCLC cell proliferation, induces apoptosis and promotes cell-cycle arrest

To assess the biological role of SNHG20 in NSCLC, we first investigated the effect of knockdown of SNHG20 on cell proliferation. MTT assays showed that cell growth was significantly impaired in si-SNHG20-transfected SPC-A1 and A549 cells (Figure 2c). Similarly, the results of colony formation assays showed that SNHG20 knockdown decreased clonogenic survival of SPC-A1 and A549 cells (Figure 2d). Ethynyl deoxyuridine (EdU) (red)/DAPI (blue) immunostaining also confirmed these results; knockdown of SNHG20 expression significantly decreased the rate of proliferation (Figure 2e).

**Figure 3:**
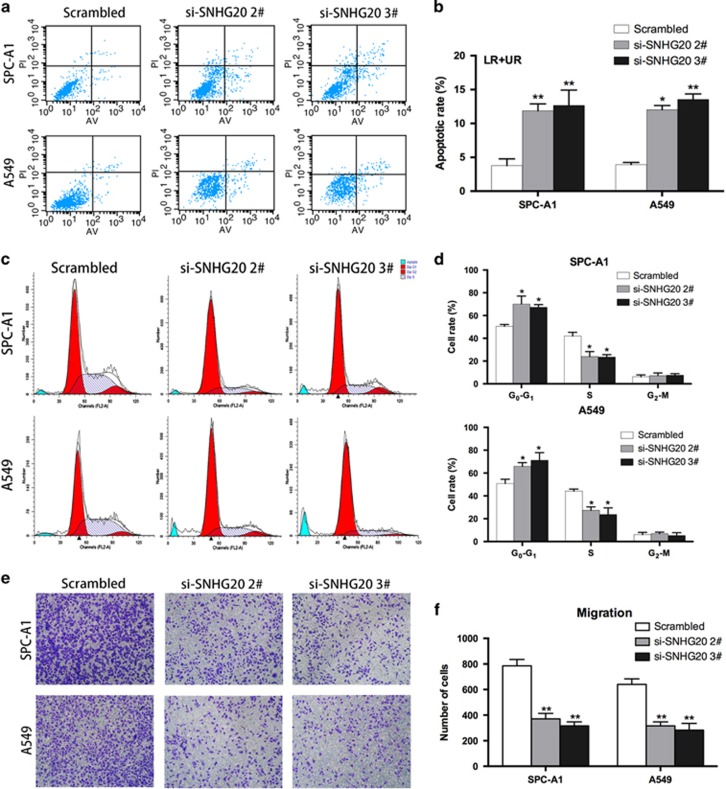
Effect of SNHG20 on NSCLC cell apoptosis, cell cycle and migration *in vitro*. (**a** and **b**) SPC-A1 and A549 cells were stained and analyzed by flow cytometry. LR, early apoptotic cells; UR, terminal apoptotic cells. (**c** and **d**) Flow cytometry showing significant decreases or increases in the proportion of cells in the S or G1 phase, respectively, when SNHG20 was silenced in SPC-A1 and A549 cells. (**e**) Transwell assays were performed to investigate changes in migratory abilities of SPC-A1 and A549 cells. Data are presented as the mean±S.E.M. All experiments were performed in triplicate with three technical replicates. *P<0.05, **P<0.01

To examine whether the effect of SNHG20 knockdown on NSCLC cell proliferation affected apoptosis or cell-cycle progression, flow cytometric analysis was performed. The proportion of apoptotic cells following treatment with SNHG20 siRNAs was significantly increased compared with that produced by scrambled control treatment (Figures 3a and b). In addition, knockdown of SNHG20 in SPC-A1 and A549 cells promoted cell-cycle arrest at the G_1_–G_0_ phase and decreased the number of cells in the G_2_–S phase (Figures 3c and d). Taken together, these data indicate that SNHG20 may drive NSCLC cell proliferation by inhibiting apoptosis and the G1–S checkpoint.

### Decreased SNHG20 expression inhibits NSCLC cell migration

To investigate the effect of SNHG20 knockdown on NSCLC cell migration, Transwell assays were performed. As shown in Figure 3e, inhibition of SNHG20 impeded the migration of SPC-A1 and A549 cells compared with controls. These data indicate that SNHG20 can promote the migratory phenotype of NSCLC cells.

### Downregulation of SNHG20 inhibits NSCLC tumorigenesis *in vivo*

**Figure 4:**
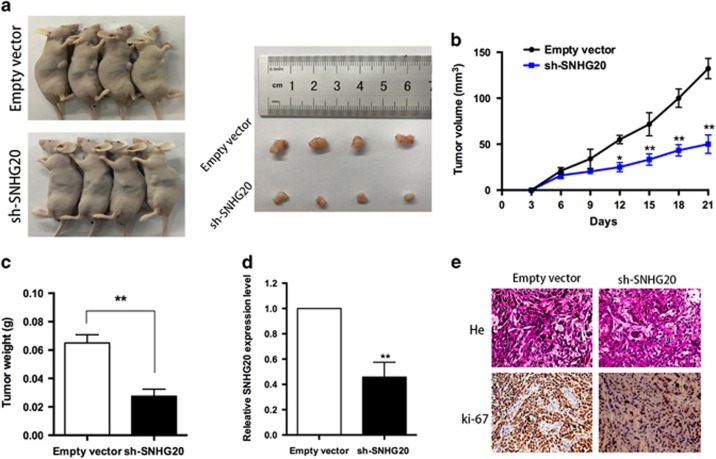
Effect of SNHG20 on NSCLC tumorigenesis *in vivo*. (**a** and **b**) Stable SNHG20-knockdown A549 cells were used for *in vivo* assays. Growth curves of tumors from two groups of mice injected with A549 cells stably transfected with sh-SNHG20 or empty vectors are shown. Tumor volumes were calculated every 3 days. (**c**) Tumor weights from the two groups are represented. (**d**) qRT-PCR was performed to detect the average expression of SNHG20 in xenograft tumors (*n*=4). (**e**) Tumors developed from sh-SNHG20-transfected A549 cells showed lower Ki-67 protein levels compared with tumors developed from control cells. Upper: hematoxylin and eosin staining; lower: immunohistochemical staining. *P<0.05, **P<0.01

To further investigate whether the level of SNHG20 expression could impact tumorigenesis *in vivo*, A549 cells stably transfected with sh-SNHG20 or empty vectors were inoculated into male nude mice. Twenty-one days after injection, all mice developed xenograft tumors at the injection site and tumor size in the sh-SNHG20 group was significantly smaller compared with that in the control group (Figure 4a). Moreover, tumor growth in the sh-SNHG20 group was significantly slower than that in the control group (Figure 4b). Additionally, the mean weight of sh-SNHG20 tumors was significantly lower compared with that in the control group (Figure 4c). Next, we used qRT-PCR analysis to show that the average expression of SNHG20 in tumor tissues was lower in the sh-SNHG20 group compared with that in the control group (Figure 4d). As shown in Figure 4e, immunohistochemistry confirmed that tumors formed from A549/sh-SNHG20 cells displayed lower-intensity Ki-67 staining compared with those formed from empty vector-transfected cells (Figure 4e). These results further confirmed that SNHG20 is involved in the development of NSCLC through its effect on NSCLC cell proliferation; the inhibition of SNHG20 expression can result in decreased NSCLC cell growth.

### SNHG20 influences P21 transcription by interacting with EZH2 in NSCLC cells

**Figure 5:**
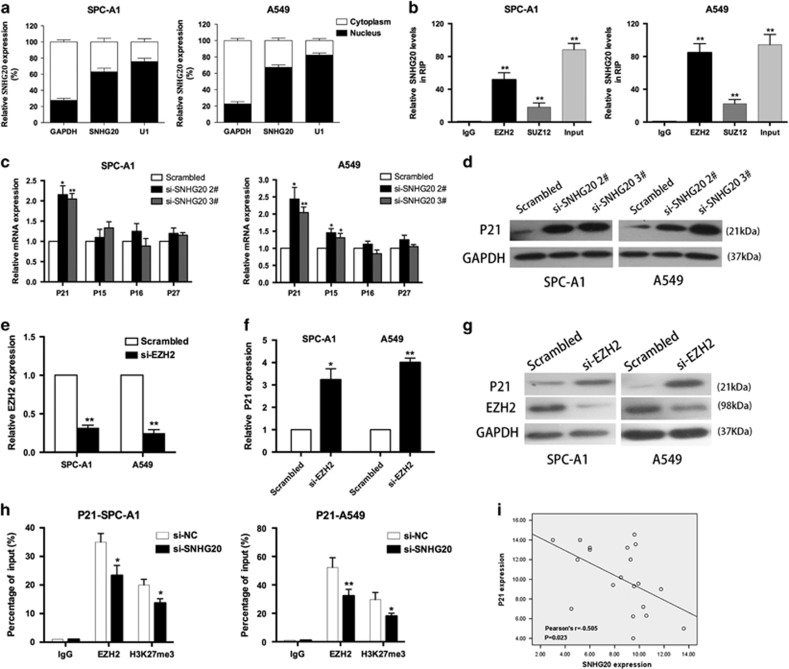
SNHG20 interacted with EZH2, and regulated P21 expression in NSCLC cells. (**a**) SNHG20 expression levels in the cytoplasm or nucleus of SPC-A1 and A549 cells were detected by qRT-PCR. GAPDH was used as a cytosol marker and U1 was used as a nuclear marker. (**b**) RNA immunoprecipitation experiments were performed in SPC-A1 and A549 cells and the coprecipitated RNA was subjected to qRT-PCR for SNHG20. SNHG20 RNA expression levels are presented as fold enrichment in EZH2 and SUZ12 immunoprecipitates relative to that of IgG. (**c**) The expression of p15, p16, P21 and p27 was determined using qRT-PCR after knockdown of SNHG20. (**d**) Western blot analysis was conducted to detect the level of P21 protein in SPC-A1 and A549 cells transfected with si-SNHG20. (**e**–**g**) qRT-PCR and western blot assays were used to detect EZH2 and P21 mRNA and protein levels in SPC-A1 and A549 cells transfected with si-EZH2. (**h**) ChIP-qRT-PCR of EZH2 occupancy and H3K27me3 binding to the P21 promoter in SPC-A1 and A549 cells treated with si-SNHG20 (48 h) or si-NC; IgG as a negative control. (**i**) Analysis of the relationship between SNHG20 expression and P21 mRNA levels (DCt value) in NSCLC patient tissue from GEO. Values are shown as the mean (S.D.) from three independent experiments. *P<0.05, **P<0.01

To explore the molecular mechanisms by which SNGH20 contributes to the phenotypes of NSCLC cells, we detected the subcellular localization of SNHG20 in NSCLC cells using fractionation assays. SNHG20 expression was higher in the nucleus than in the cytosol in both cell lines (Figure 5a), indicating that it may function as a regulator of transcription levels. Previous studies have shown that lncRNAs recruit PRC2 to the promoter of target genes and influence the expression of downstream targets.^19, 27, 28, 29^ We chose EZH2 and SUZ12, two core subunits of PRC2, to perform RNA immunoprecipitation assays. We confirmed that SNHG20 binds directly to EZH2 in SPC-A1 and A549 cells (Figure 5b).

To further investigate potential targets involved in NSCLC cell proliferation, we determined the expression of proliferation regulators, including P27, P15, P16, and P21 in SNHG20-knockdown NSCLC cells. qRT-PCR results showed that downregulation of SNHG20 significantly increased P21 expression compared with control cells (Figure 5c). In addition, clear changes in the levels of P21 protein were observed inversely correlated with the knockdown of SNHG20 in SPC-A1 and A549 cells (Figure 5d).

To determine whether SNHG20 repressed P21 expression in NSCLC cells via interaction with EZH2, we performed qRT-PCR and western blot analysis. Inhibition of EZH2 upregulated the mRNA and protein levels of P21 (Figures 5e–g). To further determine whether SNHG20 silenced P21 transcription by recruiting EZH2 to the P21 promoter region, we designed P21 primers to the promoter region and performed chromatin immunoprecipitation assays. The results showed that EZH2 could bind to the P21 promoter region, while knockdown of SNHG20 reduced EZH2 binding to P21 promoter region (Figure 5h).

Moreover, correlation analysis using 20 paired NSCLC and adjacent non-tumor lung tissues showed that SNHG20 expression was inversely correlated with P21 expression in NSCLC tissues (Figure 5i). These data indicate that SNHG20 contributes to NSCLC cell proliferation partly through the epigenetic silencing of P21 transcription by binding to EZH2; however, other possible targets and mechanisms need to be further investigated.

### Inhibition of P21 is potentially involved in the oncogenic function of SNHG20

**Figure 6:**
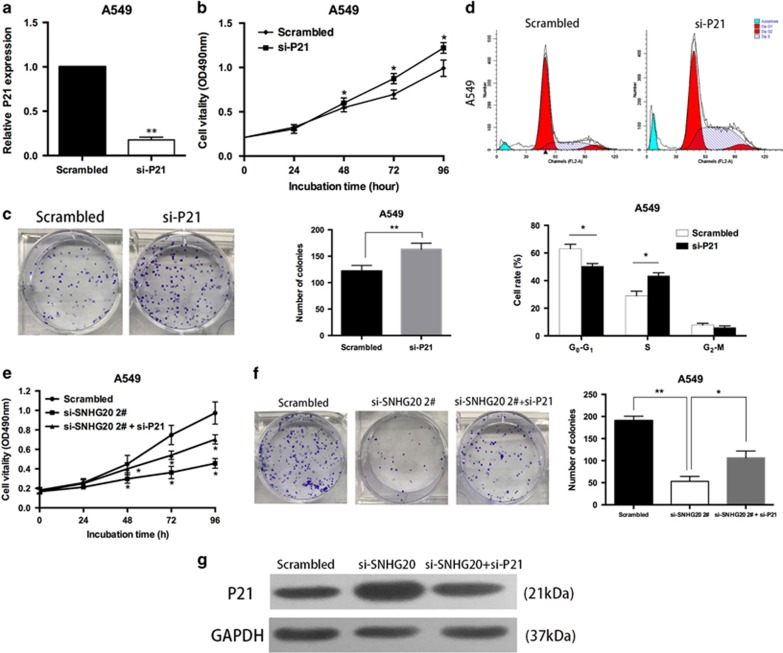
Downregulation of P21 promotes A549 cell proliferation and SNHG20 negatively regulates expression of P21. (**a**) A549 cells were transfected with si-P21. (**b** and **c**) MTT and colony formation assays were used to determine the cell viability of si-P21-transfected A549 cells. Experiments were performed in triplicate. (**d**) Flow cytometry assays were performed to analyze cell-cycle progression of A549 cells transfected with si-P21. The bar chart represents the percentage of cells in G0–G1, S or G2–M phases, as indicated. (**e** and **f**) MTT and colony formation assays were used to determine the cell viability of si-SNHG20 and si-P21 co-transfected A549 cells. (**g**) P21 expression was analyzed by western blotting. Values are shown as the mean (S.D.) from three independent experiments. *P<0.05, **P<0.01

To validate the influence of P21 on NSCLC cell proliferation, P21 expression was knocked down in A549 cells (Figure 6a). MTT and colony formation assays demonstrated that NSCLC cell viability was significantly promoted upon P21 knockdown (Figures 6b and c). Next, flow cytometry analysis showed G_0_–G_1_ cell-cycle arrest of A549 cells transfected with si-P21 (Figure 6d). These data showed that inhibition of P21 promotes proliferation and cell-cycle progression of NSCLC cells.

Moreover, we conducted rescue assays to determine whether P21 was involved in SNHG20-mediated NSCLC cell proliferation. A549 cells were co-transfected with SNHG20 and P21 siRNAs. MTT and colony formation assays indicated that the proliferation of A549 cells co-transfected with si-SNHG20 and si-P21 was increased compared with A549 cells transfected with si-SNHG20 alone (Figures 6e and f). Western blot analysis showed that cotransfection reduced upregulation of P21 induced by the depletion of SNHG20 (Figure 6g). Collectively, these data indicate that SNHG20 promotes NSCLC cell proliferation in part through the downregulation of P21.

## Discussion

Recent studies have suggested that many lncRNAs are of great importance in lung cancer, such as HOTAIR, NEAT1 and PVT1. HOTAIR promotes lung cancer cell migration and invasion by interacting with chromatin remodeling factor LSH to influence the ratio of FOXA1 to FOXA2.^30^ NEAT1 promotes NSCLC progression by functioning as a competing endogenous RNA (ceRNA) for hsa-miR-377-3p.^31^ Our previous study showed that lncRNA, LINC00152, contributes to NSCLC cell proliferation though suppression of IL24 expression, which is mediated by interaction with EZH2.^29^ However, the contribution of other dysregulated lncRNAs to NSCLC, and their functions and molecular mechanisms in NSCLC cells are poorly understood.

In the present study, we identified another lncRNA, SNHG20, which is overexpressed in NSCLC cells. The high level of SNHG20 expression in NSCLC patients was positively correlated with poor prognosis. Moreover, our results indicate that SNHG20 expression represents a predictive value and prognostic marker for patients with NSCLC. In addition to these findings, upregulation of SNHG20 was also found to predict poor prognosis in colorectal cancer and hepatocellular carcinoma.^24, 25, 26^ Moreover, loss or gain of function assays revealed that SNHG20 downregulation led to a marked inhibition of proliferation and migration *in vitro*, and suppression of tumor growth *in vivo*. Conversely, ectopic expression of SNHG20 induced malignant tumor cell behaviors. Taken together, these data indicate that SNHG20 may function as an oncogene and might play an important role in NSCLC development and progression.

Generally, lncRNAs influence cancer cell behavior by regulating the expression of target genes. In this study, we investigated the expression of important cell cycle and growth regulators after knockdown of SNHG20 in NSCLC cells, and identified P21 as a new target of SHNHG20 in NSCLC cells. lncRNAs can regulate gene expression at different levels, including chromatin modification, and transcriptional and post-transcriptional processing.^32^ To confirm the regulatory mechanism of SNHG20, we performed RNA immunoprecipitation assays and found that P21 could be regulated through histone modification mediated by SNHG20 and EZH2. P21, a cyclin-dependent kinase (CDK) inhibitor, is downregulated in a variety of cancer types,^33^ and plays critical roles in multiple cellular processes during unperturbed cell growth by directly binding to kinases related to G1/S transition, such as CyclinD/CDK4, CyclinD/CDK6 and CyclinE/CDK2.^34, 35, 36^ Here, we found P21 was a downstream regulator involved in SNHG20-mediated growth arrest in lung cancer cells. Rescue experiments indicated that inhibition of P21 potentially contributed to the oncogenic function of SNHG20.

In summary, our findings show that SNHG20 is upregulated in NSCLC tissues and that its upregulation is associated with poor prognosis for NSCLC patients. SNHG20 may promote the proliferation and migration of NSCLC cells partly through silencing P21 expression. Better understanding of SNHG20 mechanisms in the molecular etiology of lung cancer will be helpful for the development of lncRNA-based diagnostic and therapeutic agents against cancers. Our study provides a new perspective for SNHG20 acting as a non-coding oncogene in NSCLC tumorigenesis and, therefore, it is a novel early diagnostic marker and target for treatment of NSCLC. However, the other possible mechanisms by which SNHG20 participates in NSCLC cell functions remain to be fully understood.

## Materials and methods

### Tissue collection

Forty-two paired NSCLC and adjacent non-tumor lung tissues were obtained from patients who were diagnosed with NSCLC based on histopathological evaluation and had undergone surgery at Jiangsu Province Hospital between 2013 and 2015. No patients received chemotherapy or radiotherapy prior to surgery. Our study was approved by the Research Ethics Committee of Nanjing Medical University and written informed consent was obtained from all patients. All collected tissue samples were immediately snap-frozen in liquid nitrogen and stored at −80 °C until required. Clinicopathological characteristics, including advanced tumor, lymph node and metastases (TNM) staging, were recorded and are summarized in Table 1. Follow-up studies included physical examination, laboratory analysis and computed tomography if necessary. PFS was defined as the interval between the dates of surgery and recurrence; if recurrence was not diagnosed, patients were censored on the date of death or the last follow-up.

### Cell lines

Five NSCLC cell lines (PC9, SPC-A1, NCIH1975, H1299 and A549) and a normal human bronchial epithelial cell line (16HBE) were purchased from the Institute of Biochemistry and Cell Biology of the Chinese Academy of Sciences (Shanghai, China). A549, H1975 and H1299 cells were cultured in RPMI 1640 (Invitrogen, Shanghai, China); 16HBE, PC9 and SPC-A1 cells were cultured in Dulbecco’s modified Eagle’s medium (DMEM) (Invitrogen, China) supplemented with 10% fetal bovine serum (FBS), 100 IU/ml penicillin and 100 *μ*g/ml streptomycin (Invitrogen, Carlsbad, CA, USA). All cells were cultured at 37 °C in an atmosphere of humidified air, 5% CO_2_.

### RNA extraction and qPCR assays

Total RNA was extracted from tissues or cultured cells using TRIzol reagent (Invitrogen, Grand Island, NY, USA) according to the manufacturer’s instructions. Total RNA (500 ng) was reverse transcribed in a final volume of 10 *μ*l using random primers under standard conditions for the PrimeScript RT reagent Kit (TaKaRa, Dalian, China). We used SYBR Premix Ex Taq (TaKaRa, Dalian, China) to determine SNHG20 expression levels, following the manufacturer's instructions. Results were normalized to the expression of glyceraldehyde-3-phosphate dehydrogenase (GAPDH). The specific primers used are shown in Supplementary Table S1. Quantitative PCR (qPCR) and data collection were carried out on an ABI 7500 real-time PCR system (Applied Biosystems, Foster City, CA, USA). Our qRT-PCR results were analyzed and expressed relative to threshold cycle (CT) values, and then converted to fold changes.

### Cell transfection

NSCLC cells were transfected with siRNAs using Lipofectamine 2000 (Invitrogen) and incubated for 48 h prior to qPCR and western blot analyses. Three individual SNHG20 siRNAs (si-SNHG20 1#, 2# and 3#) and a scrambled negative control siRNA (si-NC) were purchased from Invitrogen. siRNA sequences are listed in Supplementary Table S1.

### Cell viability assays

A cell proliferation assay was performed with Cell Proliferation Reagent Kit I (MTT) (Roche Applied Science, Basel, Switzerland) according to the manufacturer’s instructions. SPC-A1 or A549 cells transfected with si-SNHG20 (3000 cells/well) were grown in 96-well plates. Cell viability was assessed every 24 h following the manufacturer's protocol. For the colony formation assay, a total of 500 SPC-A1 or A549 cells transfected with si-SNHG20 were placed in each well of a six-well plate and maintained in DMEM or RPMI 1640 containing 10% FBS for about 14 days, replacing the medium every 4 days. After 14 days, the colonies were fixed with methanol and stained with 0.1% crystal violet (Sigma-Aldrich, St. Louis, MO, USA), and colony formation was determined by counting the number of stained colonies. For each treatment group, wells were assessed in triplicate, and experiments were independently repeated three times.

### Flow cytometric analysis

We harvested SPC-A1 and A549 cells by trypsinization 48 h after transfection with si-SNHG20. Double staining with FITC Annexin V and propidium iodide (PI) was performed using the FITC Annexin V Apoptosis Detection Kit (BD Biosciences, Franklin Lakes, NJ, USA) according to the manufacturer's recommendations. Cells were classified into viable cells, dead cells, early apoptotic cells and apoptotic cells, and then the relative ratio of early apoptotic cells was compared with that of the control transfectant for each experiment. Cells for cell-cycle analysis were stained with PI using the CycleTEST PLUS DNA Reagent Kit (BD Biosciences) following the protocol and analyzed by FACScan flow cytometry. The percentages of cells in G0/G1, S and G2/M phases were counted and compared.

### Western blot analysis and antibodies

Cell protein lysates were separated by 10% sodium dodecyl sulfate (SDS) polyacrylamide gel electrophoresis, transferred to 0.22 *μ*m polyvinylidene fluoride membranes (Millipore, Billerica, MA, USA), and incubated with specific antibodies. ECL chromogenic substrate was used for quantification by densitometry (Quantity One software; Bio-Rad, Hercules, CA, USA). GAPDH antibody was used as a control. Anti-P21 and anti-EZH2 were purchased from Cell Signaling Technology (Boston, MA, USA).

### Cell migration assays

For the Transwell migration assays, at 48 h post-transfection, 5 × 10^4^ cells in serum-free medium were placed into the upper chamber of an insert (8 *μ*m pore size; Millipore). Medium containing 10% FBS was added to the lower chamber. After incubation for 24 h, the cells remaining on the upper membrane were removed with cotton wool. Cells that had migrated or invaded through the membrane were stained with methanol and 0.1% crystal violet, imaged and counted using an IX71 inverted microscope (Olympus, Tokyo, Japan). The experiment was performed in triplicate and repeated three times.

### Subcellular fractionation

The separation of nuclear and cytosolic fractions was performed using a PARIS Kit (Life Technologies, Carlsbad, CA, USA) following the manufacturer’s instructions.

### RNA immunoprecipitation

RNA immunoprecipitation was used to investigate whether SNHG20 could interact or bind with potential binding proteins (EZH2 and SUZ12) in SPC-A1 and A549 cells. We used the EZMagna RIP kit (Millipore) following the manufacturer’s protocol. SPC-A1 and A549 cells were lysed in complete RIP lysis buffer, and 100 *μ*l whole-cell extract was incubated with magnetic beads conjugated with antibodies that recognized EZH2, SUZ12 or control IgG (Millipore) for 6 h at 4 °C. Then, the beads were incubated with 0.1% SDS/0.5 mg/ml proteinase K for 30 min at 55 °C to remove proteins. Finally, immunoprecipitated RNA was subjected to qRT-PCR analysis using specific primers to demonstrate the presence of SNHG20.

### Chromatin immunoprecipitation assays

A549 and SPC-A1 cells were treated with formaldehyde and incubated for 10 min to generate DNA–protein crosslinks. Cell lysates were then sonicated to generate chromatin fragments of 200–300 bp and immunoprecipitated with EZH2 or H3K27me3-specific antibodies (Millipore) or IgG as control. Precipitated chromatin DNA was recovered and analyzed by qRT-PCR.

### Tumor formation assay in a nude mouse model

A549 cells were stably transfected with sh-SNHG20 or empty vector and cells were collected at a concentration of 2 × 10^7^ cells/mL and 0.1 ml was subcutaneously injected into either side of the posterior flank of male BALB/c nude mice (4–5 weeks old). Mice were purchased from Shanghai Experimental Animal Center of the Chinese Academy of Sciences (Shanghai, China). Tumor volumes and weights were measured every 3 days and tumor volumes were calculated using the following equation: *V*=0.5 × *D* × *d*2 (*V*, volume; *D*, longest diameter; *d*, diameter perpendicular to the longest diameter). At 21 days after injection, mice were killed, and the subcutaneous growth of each tumor was examined. Primary tumors were excised and tumor tissues were used to perform qPCR analysis of SNHG20 levels and immunostaining of Ki-67 protein. This study was carried out in strict accordance with the recommendations in the Guide for the Care and Use of Laboratory Animals of the National Institutes of Health. The protocol was approved by the Committee on the Ethics of Animal Experiments of the Nanjing Medical University.

### Immunohistochemistry

Primary tumors were immunostained for Ki-67 as previously described.^28^

### Statistical analysis

All statistical analyses were performed using SPASS 20.0 software (IBM). The significance of differences between groups was estimated by Student’s *t*-test, Wilcoxon’s test or the *χ*^2^ test. Disease-free survival (DFS) and OS rates were calculated by the Kaplan–Meier method with the log-rank test applied for comparison. Survival data were evaluated using univariate and multivariate Cox proportional hazards models. Variables with a value of *P*<0.05 in univariate analysis were used in subsequent multivariate analysis on the basis of Cox regression analyses. Kendall Tau-b and Pearson correlation analyses were conducted to investigate the correlation between SNHG20 and P21 expression. *P*-values less than 0.05 were considered statistically significant.

## Figures and Tables

**Table 1:** Correlation between SNHG20 expression and clinicopathological characteristics of NSCLC patients (*n*=42)

**Characteristics**	**SNHG20 low number of case (%)**	**High number of case (%)**	***χ***^**2**^**test (*****P***-**value)**
*Age (years)*			
>65	12	10	0.758
≤65	9	11	
			
*Gender*			
Male	11	13	0.756
Female	10	8	
			
*Histologic subtype*			
Squamous cell carcinoma	9	13	0.354
Adenocarcinoma	12	8	
			
*TNM stage*			
Ia+Ib	11	2	0.008*
IIa+IIb	6	8	
IIIa	4	11	
			
*Tumor size*			
≤5 cm	14	5	0.012*
>5 cm	7	16	
			
*Lymph node metastasis*			
Negative	15	5	0.005*
Positive	6	16	
			
*Smoking history*			
Smokers	12	15	0.526
Never smokers	9	6	

*P<0.05
